# Pseudohypertrophy Medial Gastrocnemius Muscle due to Chronic Radiculopathy

**DOI:** 10.5334/jbsr.3902

**Published:** 2025-03-20

**Authors:** Rob Vernelen, Filip M. Vanhoenacker

**Affiliations:** 1 AZ Sint‑Maarten Mechelen and KU Leuven, Belgium; 2 AZ Sint‑Maarten Mechelen and University (Hospital), Antwerp/Ghent, Belgium

**Keywords:** Pseudohypertrophy, Medial gastrocnemius, Chronic radiculopathy, Ultrasound, MRI

## Abstract

*Teaching point:* Denervation pseudohypertrophy of the medial gastrocnemius muscle is an uncommon cause of calf swelling that may be secondary to chronic radiculopathy.

## Case History

**Figure 1:**
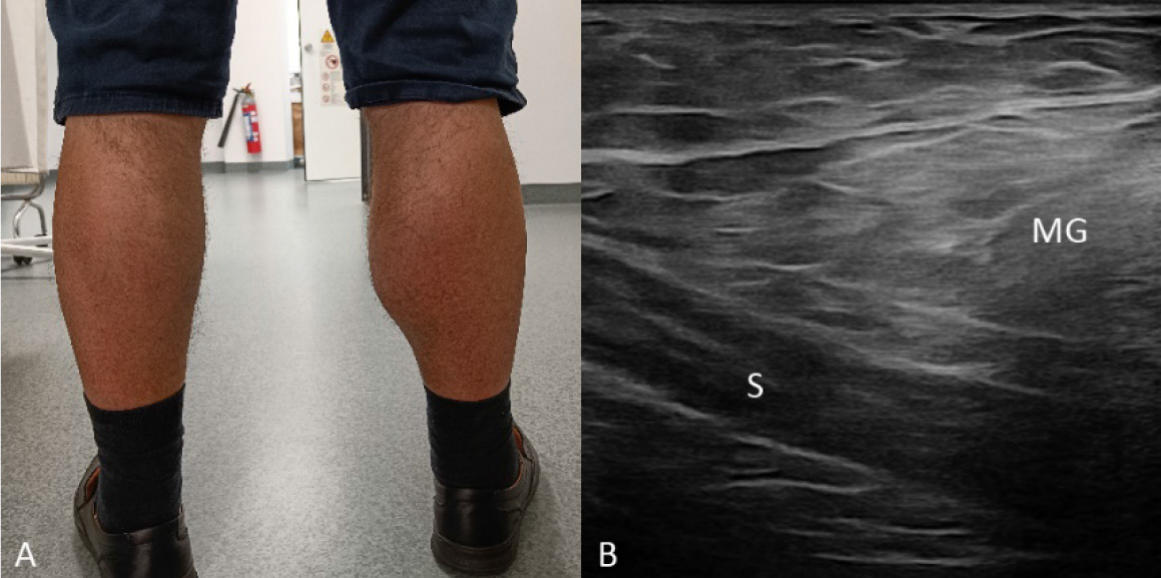
(**A**) Clinical image: Swollen right calf. (**B**) US: Enlargement and heterogeneous hyperechogenicity of the medial gastrocnemius muscle (MG) compared with normal soleus muscle (S).

**Figure 2:**
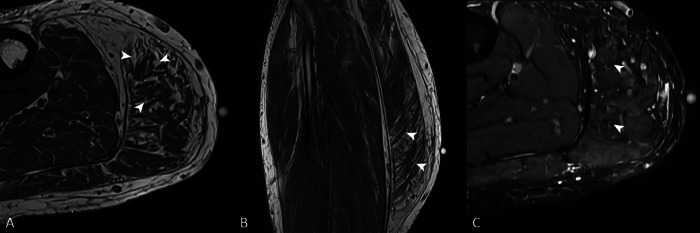
(**A**) Axial and (**B**) sagittal T1‑WI show enlargement of the medial gastrocnemius and intramuscular streaks of hyperintense signal (arrowheads). (**C**) Fat‑saturated T2‑WI show marbled appearance (arrowheads) consistent with partial fatty degeneration.

**Figure 3:**
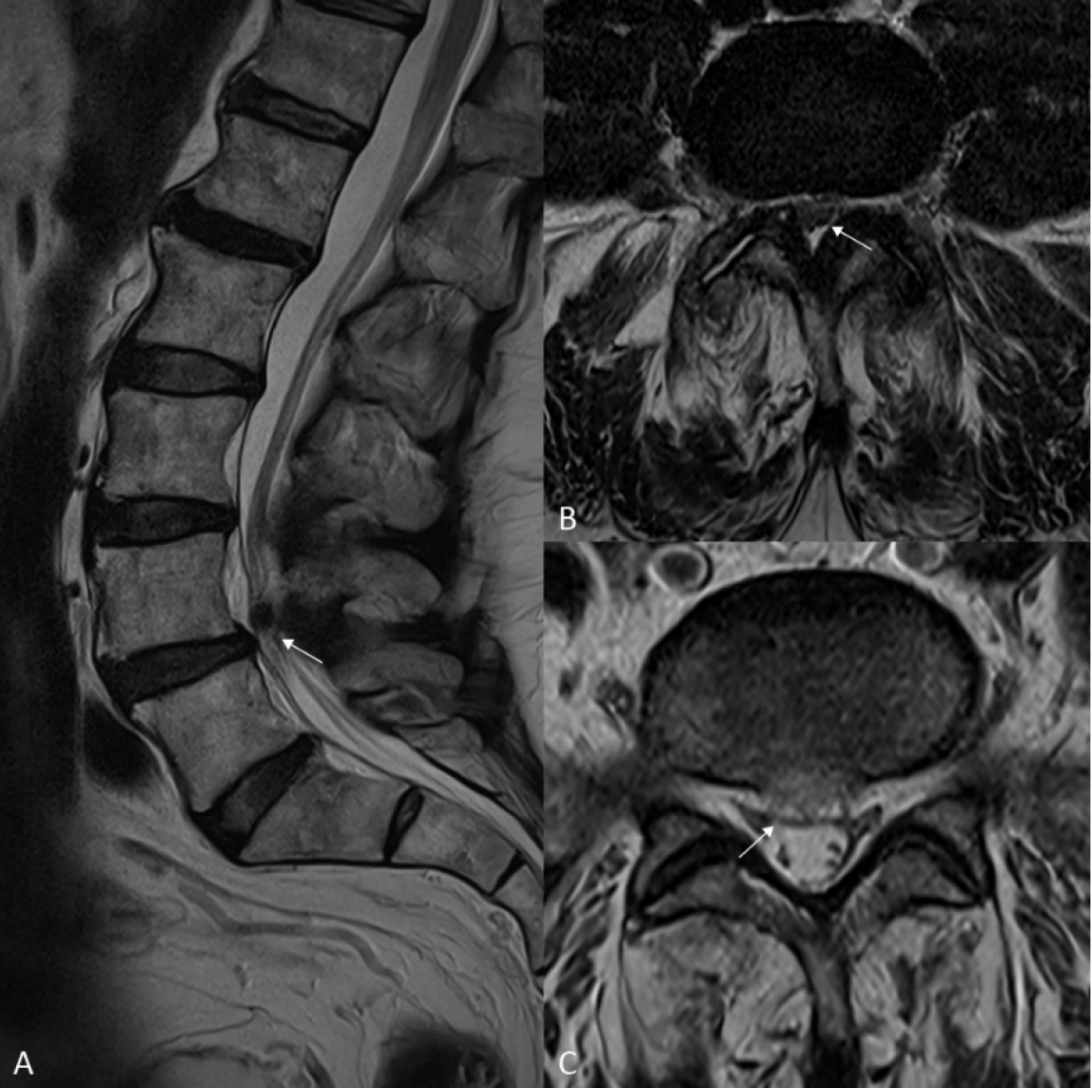
(**A**) Sagittal and (**B**) axial T2‑WI showing severe aquired spinal stenosis at L4‑L5 (arrows). (**C**) Axial T2‑WI showing a right sided disc protrusion at L5‑S1 (arrow).

A 62‑year‑old male presented with a swollen right calf and chronic pain during weight‑bearing (Figure 1A). Ultrasound (US) revealed enlargement and heterogeneous hyperechogenicity of the medial gastrocnemius muscle (MG) compared with the normal echotexture of the soleus muscle (S) (Figure 1B). Magnetic resonance imaging (MRI) confirmed enlargement of the MG and showed intramuscular streaks of hyperintense signal on axial and sagittal T1‑weighted images (WI) (Figure 2A–B), isointense to fat on fat‑saturated T2‑WI (Figure 2C) with a marbled appearance (Figure 2, arrowheads) consistent with partial fatty degeneration. The patient was known to have degenerative lumbosacral disease with severe acquired spinal stenosis at the L4–L5 level (MRI; Figure 3A−B, arrows) without recognizable rootlets and complete effacement of cerebrospinal fluid space, but sparing of the epidural fat posteriorly (Schizas grade C) as well as a right‑sided disc protrusion at L5–S1 (Figure 3C, arrow) with root contact at S1.

On the basis of the combination of the clinical presentation and the imaging findings, the diagnosis of denervation pseudohypertrophy was suggested.

## Comments

Denervation pseudohypertrophy results from partial denervation and has been reported in various conditions, including diabetic neuropathy, peripheral nerve injury, and spina bifida [1], or as in this case, it can be due to chronic radiculopathy with continuation of physical exercise. The gastrocnemius muscle is innervated by the anterior branches of the S1 and S2 spinal nerves through the tibial nerve.

Muscle denervation typically progresses from muscle edema in the acute phase to atrophy and fatty replacement over time. Atrophy and fatty infiltration are two separate processes occurring simultaneously. Rarely, pseudohypertrophy occurs, characterized by a combination of true muscle hypertrophy with interspersed fatty infiltration [1]. Axonal damage in chronic radiculopathy leads to reduced muscle fiber size, stimulating mesodermal cell differentiation into lipocytes, resulting in fat deposition in the affected compartment. Persistent exercise and stretching, however, can induce hypertrophy in partially denervated muscle fibers and promote re‑innervation of denervated fibers in the same compartment.

True muscle hypertrophy is caused by increased physical workload on both partially denervated and re‑innervated fibers associated with abnormal spontaneous bioelectrical activity. Whenever denervation pseudohypertrophy of the calf muscles is encountered on ultrasound or MRI, review of a clinical history of chronic radiculopathy and MRI of the lumbar spine is indicated to allow a precise etiopathogenetic diagnosis.

By the time the condition is clinically apparent, denervation has typically progressed to a stage where neural injury is largely irreversible [1]. Treatment is limited to managing the underlying radiculopathy to prevent further damage.
